# Health-related quality of life and associated factors among adult podoconiosis patients in Debre Elias district Northwest, Ethiopia

**DOI:** 10.1371/journal.pntd.0010673

**Published:** 2022-09-02

**Authors:** Abraham Abebaw, Asmamaw Atnafu, Nigusu Worku, Asebe Hagos

**Affiliations:** 1 Debre Elias district health office, East Gojjam Zone, Amhara Regional National State, Bahir Dar, Ethiopia; 2 Department of Health Systems and Policy, Institute of Public Health, College of Medicine and Health Sciences, University of Gondar, Gondar, Ethiopia; University of Buea, CAMEROON

## Abstract

**Background:**

Podoconiosis is endemic non-filarial elephantiasis and non-infective neglected tropical disease. It has a wide impact on the physical, social and psychological aspects of the well-being of a person. However, limited information is available about the disease burden on health-related quality of life and associated factors in Ethiopia.

**Objective:**

This study aimed is to determine health-related quality of life and associated factors among adult podoconiosis patients in Debre Elias district, Northwest, Ethiopia.

**Methods:**

A community-based cross-sectional study was conducted from February 1 to March 30, 2020 in the Debre Elias district. A multi-stage stratified; systematic random sampling technique was employed to select 403 podoconiosis patients. The data were collected through an interviewer-administered questionnaire. Data were entered into Epi data version 4.6 and exported to STATA version 14 for analysis. After the assumption check for the linear regression model, simple and multiple regression was done to see the association between the predictor and outcome variables. Predictor variables that had p-value <0.2 at simple linear regression were taken into multiple linear regression. β coefficient with 95% CI and p-value of <0.05 was considered as statistically significant variables in multiple linear regression analysis.

**Result:**

The overall mean quality of life score among podoconiosis patients was 61.93±17.14. The mean quality of life score for the physical, psychological, social, and environmental domains were 75.57±21.86, 60.43±18.58, 30.34±10.46, and 81.38±22.77 respectively. Foot care had a statically significant association with all domains. Higher quality of life podoconiosis patients was associated with foot care. Lower quality of life was associated with the presence of anxiety, advanced stage of the disease, and frequent adenolymphangitis attack.

**Conclusion:**

Social and psychological domains of quality of life were lowest as compared to physical and environmental domains of quality of life. Early medical treatment, psychosocial support, and home-based foot care should be encouraged to improve the quality of life in podoconiosis patients.

## Author summary

Podoconiosis: is a disease that affects the lymph vessels of the lower extremities and is caused by a person’s foot exposed to minerals found in red clay soil. Podoconiosis has been identified in 32 countries and globally 4 million people are affected with this disease. The disease is characterized by prominent swelling) of the lower extremities, it can be prevented through keeping good foot hygiene, wearing clean socks and shoes daily and covering floor in the home to avoid contact with irritant soil. Patients with podoconiosis significantly challenged by physical disability, poor mental health and social discrimination. This study identified quality of life and associated factors among adult podoconiosis patients in Debre Elias district Northwest, Ethiopia. Specifically, we addressed quality of life score for physical, psychological, social and environmental domain. We found from this study foot care had statically significant association with all domains. Lower quality of life was associated with presence of anxiety, advanced stage of the disease and frequent adenolymphangitis attack. Social and psychological domains of quality of life were lowest as compared to physical and environmental domains of quality of life. Therefore, this study encouraged early medical treatment, home-based foot care and psychosocial support to improve quality of life in podoconiosis patient.

## Introduction

Podoconiosis is a non-filarial elephantiasic disease that compromises the function of the lymphatic vessels of the lower extremities’ [1,2]. It is the second most common cause of neglected tropical lymphedema after lymphatic filariasis. The disease is characterized by prominent swelling) of the lower extremities, which leads to disfigurement and disability [3–5]. Based on existing evidence, the most accepted cause of podoconiosis is that of mineral particle-induced inflammation on a background of genetic susceptibility [6].

Podoconiosis has been identified in 32 countries and globally 4 million people are affected with podoconiosis [7] mainly in tropical Africa, Central and South America, and Southeast Asia. The global prevalence of podoconiosis varies from 8.08% in Cameroon to 2.51% in Tanzania [8] with in the podoconiosis affected countries.

The greatest burden of podoconiosis globally is assumed to occur in Ethiopia [9]. According to 2015 estimation, over 1.5 million cases of podoconiosis exist in Ethiopia [6,10]. The disease is endemic in 345 districts; mainly the three regions (South Nation, Nationalities and Peoples, Oromia and Amhara) contributed 99% of the cases [6] with the prevalence of the disease ranging from 9.1% in Illubabor Zone, Oromia Region [11] to 2.8% in Gulliso District, Oromia region [12], whereas 3.3% in Debre Elias, East Gojjam Zone, Amhara region [13].

The disease has imposed a huge impact on the social, psychological, and economic burden in affected individuals [14,15]. These patients face significant physical disability, psychological comorbidity and experience frequent episodes of systemic illness due to acute adenolymphangitis attacks (ALA). Different studies showed that patients with podoconiosis disease have a lower quality of life [16–18].

Quality of life is a broad and complex evaluation of an individual’s current life circumstances in the context of the culture in which they live and the values they hold [19]. When the quality of life is considered in the context of health and disease, it’s commonly referred to as health-related quality of life (HRQoL). HRQoL can explore the physical, social, and psychological impact of the disease [20,21]. HRQoL is increasingly acknowledged as a valid and appropriate indicator of service need and intervention outcomes in public health research and practice [22].

A study conducted in Cameroon showed that people with lower limb lymphedema like podoconiosis were more predisposed to develop mental disorder as compared to healthy individuals which suggest that the psychological and social domain of their HRQoL was more affected and resulted in poor HRQoL [18]. The presence of the disease had a negative impact on HRQoL. It reduces the physical, psychological, environmental, and social domains of health-related quality of life of podoconiosis patients [17,18].

Evidence in Ethiopia showed that podoconiosis related social stigma had a major impact on the psychological wellbeing and social health of patients. The disease is one of the commonest causes of stigmatization among community members and it leads to the social exclusion of individuals and their families. Patients are banned from social events like weddings, funerals, schools, local meetings, and churches [14,23]. A study conducted in southern Ethiopia shows that podoconiosis patients faced stigma in their daily interaction. Unwillingness to marry with podoconiosis patient, avoiding physical contact, spitting on patients, pinching nose when walking past patients at a distance was mentioned by patients as a manifestation of stigma. Most patients described it as ’the worst disease’ mainly due to its negative social and psychological consequences resulting in poor quality of life [24,25].

Deformity and disability problems related to most neglected tropical diseases like podoconiosis finally lead to poor mental health in affected individuals. People with podoconiosis are found to have a higher score of mental distress. The disease affects the psychological and social domains negatively and that results in poor health-related quality of life in podoconiosis patients as compared to healthy individuals [26].

Even though podoconiosis has a negative impact on patients’ HRQoL, the existing studies mainly focus on determining the prevalence, risk factors, geographical distribution, and disease-related stigma. However, little information is available about the health-related quality of life and associated factors among podoconiosis patients in Ethiopia. Therefore, this study aimed to determine the magnitude of health-related quality of life and associated factors among adult podoconiosis patients.

## Materials and methods

### Ethics statement

Ethical clearance was obtained from the Ethical Review Committee of the Institute of Public Health, College of Medicine and Health Sciences, University of Gondar (Reference No, HSP /8768/12). Permission letter was obtained from Debre Elias district health office and selected kebeles. During data collection, study participants were participated voluntarily and asked for consent and were signed written agreements on consent forms. Before starting the interview all the study participants were assured that the data was anonymous, names or any personal identifiers were not recorded. The participants were informed that they have the right to interrupt the interview at any moment.

### Study setting and area

A community-based cross-sectional study was conducted in Debre Elias district Northwest, Ethiopia from February 1 to March 30, 2020. The district is divided into 4 urban and 17 rural kebeles (the lowest administrative unit). The majority (84%) of the population resides in rural areas [27]. Additionally, the district has 4 public health centers and 17 health posts with more than 196 health professionals including health extension workers and administrative staff [28].

### Population

All clinically confirmed adult podoconiosis patients who live in the selected kebeles were the study population. Whereas, podoconiosis patients who live less than 6 months in the selected kebeles, patients with other conditions like, congenital physical disability, blindness, and pregnancy were excluded from the study.

### Sample size determination and sampling procedure

The sample size was determined using single population mean formula (**(n = (Zα/2)**^**2**^**σ**^**2**^**/d**^**2)**^) with an assumption of Z_(*a/2)*_ at 95% Confidence Level (CL = 1.96, standard deviation (*σ* = 17.13), 5% of the mean of margin of error *(d* = 2.15 [17], 10% non- response rate and design effect = 1.5%. Therefore, the final calculated sample size was 403.

A two-stage stratified sampling method was used to select participants from rural and urban kebeles. Two urban and five rural kebeles were selected using a simple random sampling technique, then the sample was proportionally allocated to each kebeles based on the number of patients. Finally, a systematic random sampling technique was employed to select the study participant at each stratum/kebele based on the health post list of the patient.

### Data collection tools and procedures

Health-related quality of life was assessed by the WHOQoL-BREF questionnaire. Data were collected through face-to-face interviews. The questionnaire was developed through adoption from the validated instrument of short version WHOQoL-BREF [29]. The WHOQoL-BREF is a 26-item instrument consisting of four domains: physical health (7 items), psychological health (6 items), social relationships (3 items), and environmental health (8 items); it also contains quality of life and general health items that measure the overall perception of quality of life and health status. The four domain scores denote an individual’s perception of the quality of life in each particular domain. Additionally, socio-demographic questionnaire also adopted from different literature [17,30–32].

Each item of the WHOQoL-BREF was scored from 1 to 5 on a response scale, which is stipulated as a five-point ordinal scale item. The four domain scores and two items are examined separately: question 1 asks about an individual’s overall perception of quality of life and question 2 asks about an individual’s overall perception of his or her health. Domain scores are scaled in a positive direction (i.e., higher scores denote the higher quality of life and lower scores denote the lower quality of life). But Some facets (pain and discomfort, negative feelings, bodily image, and appearance) are not scaled in a positive direction these questions are recoded (1;5, 2;4,3;3,4;2 and 5;1) than mean score of items within each domain is used to calculate the domain score and each domain mean score is transformed to a 0–100 scale by using this formula:

$$\text{Transformed}\;\text{scale}=\left(\text{Actual}\;\text{raw}\;\text{score}-\text{lowest}\;\text{possible}\;\text{score}\right)/\text{possible}\;\text{raw}\;\text{score}\;\text{range}*100$$

[33].

Where more than two items are missing from the domain, the domain score should not be calculated (except domain 3, where the domain should only be calculated if < 1 item is missing). Then the overall HRQoL was calculated by summing up each transformed domain score and divided by four (number of domains) [29].

The questionnaire was first prepared in English then translated to Amharic (local language) and back to English to check its consistency. Six BSc nurses for data collectors and two BSc health officers for supervisors were recruited. Two days of training was given on data collection procedures, and processes for consistency and completeness of the data collected. The tool of the study was pre-tested by using 5% of the total sample size from 21 podoconiosis patients in the Machakel district, which was out of the study area and a necessary amendment and correction was made. During the data collection process, the supervisor checked the data accuracy, consistency, and completeness on daily basis.

### Data processing and analysis

All completed questionnaires were manually checked for completeness and consistency of responses. In line with this, data were coded and entered in Epidata version 4.6 and exported to STATA version 14 for analysis. A reliability test (Cronbach alpha) was performed to check the reliability of the questionnaire items and domains. Negatively framed questions were transformed into positively framed questions. The raw and transformed score was done for the outcome variables. Descriptive statistics were used to summarize the overall score of HRQoL. Linear regression model assumptions tests, such as normality, linearity, independence, homoscedasticity, and multi-collinearity were checked. Simple and multiple linear regressions were done to see the association between the predictor and the outcome variables. Predictor variables that had a p-value < 0.2 in the simple linear regression were taken into multiple linear regressions. β coefficient with 95% CI and P-value of <0.05 was considered statistically as a significant variable in multiple linear regression analysis.

## Results

### Sociodemographic and economic characteristics of respondents

In this study, a total of 403 podoconiosis patients were participated with a response rate of 98.76%. Among the study participants, 62% were male, 66.3% were married, 70.3% were farmers, and 95.2% were rural residents. The mean age of the study participants was 44.4 ± 6.2 years and 33.4% of study participants were poor in wealth status (Table 1).

**Table 1:** Demographic and socio-economic characteristics of study participants at Debre Elias district, Northwest, Ethiopia in 2020(n = 398).

Variable	Description	Frequency (%)	Mean ± SD
Sex	Male	247 (62.1%)	
	Female	151 (37.9%)	
Age			44.44± 6.2
Residence.	Urban	19 (4.7%)	
	Rural	379 (95.2%)	
Family size			4.62 ±1.75
Present of employee	Absent	367 (92.2%)	
	Present	31 (7.8%)	
Ethnicity	Amhara	398 (100%)	
Educational status	Unable to read	271 (68.1%)	
	Only read and write	113 (28.4%)	
	Primary school	13 (3.3%)	
	Secondary school	1 (0.3%)	
Religion	Orthodox Christian	390 (98.0%)	
	Muslim	6 (1.5%)	
	Catholic	2 (0.5%)	
Marital status	Single	26 (6.5%)	
	Married	264 (66.3%)	
	Divorced	48 (12.1%)	
	Widowed	59 (14.8%)	
	Others	1 (0.3%)	
Occupation	Government employee	5 (1.3%)	
	Housewife	77 (19.3%)	
	Merchant	10 (2.5%)	
	Farmer	280 (70.3%)	
	Daily laborer	25 (6.3%)	
	Others	1 (0.2%)	
Wealth index score	Poor/1st quintiles	133 (33.4%)	
	Medium/2nd quintiles	133 (33.4%)	
	Rich/3rd quintiles	132 (33.2%)	

### Medical-related characteristics of respondents

More than one-third (35.6%) of the patients had stage 3 podoconiosis, and 7.2% had comorbidities. About 60.3% of the study participants developed podoconiosis related depression. Moreover, more than half (56.2%) of the study participants had not received medical care for podoconiosis disease, and the mean duration of podoconiosis was found to be 21.12 ± 9.9 years. The study indicated that near to three-forth (71.6%) of participants practiced foot care (washing of their foot and apply antiseptic solution and bandage on their foot) at least one times per week. Among those who practiced foot care, the mean frequency of foot care per week was 1.12±1.36. (Table 2).

**Table 2:** Medical-related characteristics of study participants at Debre Elias district, Northwest, Ethiopia 2020(n = 398).

Variable	Description	Frequency (%)	Mean ±SD
Duration of disease			21.12 ± 9.9
Stage of the disease	Stage 2	120 (30.2%)	
	Stage 3	142 (35.7%)	
	Stage 4	99 (24.9%)	
	Stage 5	37 (9.3%)	
Frequency of ALA per year			5.98 ± 4.62
Medical treatment	Yes	174 (43.7%)	
	No	224 (56.3%)	
Other co morbidities	Yes	29 (7.3%)	
	No	369 (92.7%)	
Depression	Absent	158 (39.7%)	
	Present	240 (60.3%)	
Anxiety	Anxiety	221 (55.5%)	
	Present	177 (44.5%)	
Social dysfunction related to podo	Absent	120 (30.2%)	
	Present	278 (69.8%)	
Frequency of Footcare per week			1.12 ±1.36
Shoe wearing	No	53 (13.3%)	
	Yes	345 (86.7%)	

### Self-rated perceived quality of life and health satisfaction

Study participants were asked to provide their perception of their health-related quality of life and health status. Accordingly, 187 (46.98%) of the participants rated their quality of life as poor and 34.42% were dissatisfied with their current health status (Table 3).

**Table 3:** Self-rated perceived quality of life and health satisfaction the participants at Debre Elias district Northwest Ethiopia, 2020.

Self-rated perceived quality of life		Self-rated perceived health satisfaction	
Response	Frequency (%)	Response	Frequency (%)
Very poor	21 (5.3%)	Very dissatisfied	25 (6.28%)
Poor	187 (46.9%)	Dissatisfied	137 (34.42%)
Neither poor nor good	55 (13.8%)	Neither satisfied nor dissatisfied	166 (41.71%)
Good	134 (33.7%)	Satisfied	62 (15.58%)
Very good	1 (0.2%)	Very satisfied	8 (2.01%)

### Health-related quality of life

The transformed mean score of the overall HRQoL of the study participants was found to be 61.93± 17.14. Among the four domains, participants scored highest (81.38±22.77) and lowest (30.34 ±10.46) health-related quality of life for environmental and social domains respectively. The internal reliability of the tool was measured using Cronbach’s alpha coefficient. The overall HRQoL-BREF score was α = 0.96 it was higher than the accepted value of 0.7 [34,35]. Similarly, all domains had a higher score of Cronbach’s alpha coefficient (Table 4).

**Table 4:** HRQoL domain score of study participants at Debre Elias district, Northwest Ethiopia, 2020 (n = 398).

Variable	Mean±SD	The reliability coefficient (Cronbach’s alpha)
Overall score of WHOQoL-BREF	61.93 ± 17.1	0.96
Physical domain score	75.57 ± 21.9	0.91
Psychological domain score	60.43 ± 18.6	0.90
Social domain score	30.34 ± 10.5	0.76
Environmental domain score	81.38 ± 22.8	0.89

### Factors associated with overall HRQoL among the study participants

In this study, about 71.84% of the variation in overall health-related quality of life in the study area is explained by family size, residence, marital status, and frequency of ALA, foot care, stage of disease, and wealth status. A higher score of overall HRQoL of life was associated with being rural residents, foot care, and shoe wearing, whereas the low health-related quality of life was associated with family size, marital status, frequency of ALA, stage of disease, and poor wealth status.

Participants living in rural area, the overall HRQoL score was 6.64 (β = 6.64, 95% CI = 2.14, to 11.14) times higher as compared with urban residents. Patients who were single and divorced had 9.30 and 3.77 (β = -9.30, 95% CI = -14.04, to -4.56-, β = -3.77, 95% CI = -7.00, to -.545) times less overall HRQoL score as compared to married patients respectively. With an increase family size by one, the overall HRQoL of podoconiosis patients was decreased by 0.74 scores (β = -.74, 95% CI = -1.45, to -.03). When the frequency of an acute ALA increases by one the overall quality of life of podoconiosis patient decreased by 0.55 scores (β = -.55, 95% CI = -0.89, to -0.21). Patients who had stage 3, stage 4 and stage 5 podoconiosis disease had 5.02, 8.37 and 10.58 (β = -5.02, 95% CI = -7.31, to -2.74, β = -8.37, 95% CI = -11.78, to -4.99, β = -10.58, 95% CI = -15.36, to -5.80) times less overall quality of life score as compared to stage two patients respectively. When the foot care increases by one day per week, the overall quality of life of podoconiosis patient was increased by 2.90 scores (β = 2.90, 95% CI = 1.91 to 3.90).

Regarding wealth status, patients who were poor and medium status had 6.37 and 2.64 (β = -6.37, 95% CI = -9.32 to -3.42, β = -2.64, 95% CI = -5.03 to -0.25) times less overall quality of life score compared to rich patients respectively (Table 5).

**Table 5:** Multiple linear regression analysis of variables associated with overall HRQoL among patients with podoconisis at Debre Elias district, Northwest, Ethiopia in 2020.

Variables Category		Coef.	P>|t|	[95% CI}
Age		-.08	0.095	-.19.01
Sex	Male	-	-	-
	Female	-.84	0.437	-2.97 1.28
Family size		-.74	**0.039**	-1.45 -.03
Residence	Urban	-	-	-
	Rural	6.64	**0.004**	2.14 11.14
Education	secondary school	-	-	-
	Unable to read and write	-4.39	0.618	-21.67 12.89
	Only read and write	-4.40	0.615	-21.61 12.80
	Primary school	-1.53	0.865	-19.30 16.22
Marital status	married	-	-	-
	Single	-9.30	**<0.001**	-14.04–4.56
	Divorced	-3.77	**0.022**	-7.00 -.545
	Widowed	-2.14	0.188	-5.34 1.05
	Others	-8.08	0.367	-25.66 9.50
Duration of disease		-.09	0.14	-.22.03
Frequency of ALA		-.55	**<0.001**	-.89 -.21
Stage of the disease	Stage 2	-	-	-
	Stage 3	-5.02	**<0.001**	-7.31–2.74
	Stage 4	-8.37	**<0.001**	-11.76–4.99
	Stage 5	-10.58	**<0.001**	-15.36–5.80
Medical Rx	No	-	-	-
	Yes	-.11	0.921	-2.34 2.11
Co-morbidity	No	-	-	-
	Yes	-.62	0.731	-4.22 2.9
	Poor	-	-	-
	Good	.065	0.962	-2.65 2.78
Depression	Absent	-	-	-
	Present	-.59	0.542	-2.52 1.33
Anxiety	No	-	-	-
	Yes	-2.17	0.251	-5.89 1.54
Social disfunction	Absent	-	-	-
	Present	-.91	0.500	-3.57 1.74
Foot care		2.90	**<0.001**	1.91 3.90
Shoe wearing	No	-	-	-
	Yes	5.84	**<0.001**	2.80 8.88
Wealth index	Rich	-	-	-
	Poor	-6.37	**<0.001**	-9.32–3.42
	Medium	-2.64	**0.030**	-5.03 -.25
_cons		76.20	**<0.001**	57.35 95.06

### Factors associated with each domain of HRQoL among the study participants

Linear regression analysis was conducted separately to assess the predictor variables for each domain of health-related quality of life among podoconiosis patients (Table 6).

**Table 6:** Multiple linear regression analysis of variables associated with each domain of HRQoL among patients with podoconiosis at Debre Elias district, Northwest, Ethiopia in 2020.

Variables	Physical		Environmental		psychological		Social	
	β	95% CI	β	95% CI	Β	95% CI	Β	95% CI
Age(years)	-.15	-.30 to -.01**	-.12	-.29 to .04	-.04	-.15 to .07	-.03	-.12 to .04
Sex								
Male	-	-	-	-	-	-	-	-
Female	-2.88	-5.97 to .20	.30	-3.18 to 3.79	-1.14	-3.46 to 1.17	.35	-1.36 to 2.07
Family size	-1.25	-2.27 to -.22**	-0.47	-1.62 to .68	-0.97	-1.75 to -.21**	-0.27	-.84 to .29
Residence								
Rural	12.17	5.65 to 18.70**	5.00	-2.37 to 12.37	5.64	.74 to 10.55**	3.75	.11 to 7.38**
Urban	-	-	-	-	-	-	-	-
Educational status								
Unable to read	-5.37	-30.46 to 19.71	3.03	-25.28 to 31.35	-12.82	-31.66 to 6.01	-2.41	-16.36 to 11.54
Only read and write	-6.13	-31.11 to 18.85	5.07	-23.12 to 33.27	-13.20	-31.96 to 5.55	-3.37	-17.27 to 10.51
Primary school	-1.61	-27.41 to 24.18	7.30	-21.80 to 36.42	-9.25	-28.62 to 10.11	-2.59	-16.94 to 11.75
Secondary and above	-	-	-	-	-	-	-	-
Marital status								
Single	-8.6	-15.48 to -1.71**	-12.48,	-20.25 to -4.71**	-11.90	-17.07 to -6.74**	-4.21	-8.04 to -.38**
Divorced	-1.12	-5.81 to 3.56	-5.90	-11.28 to -.69**	-4.77	-8.29 to -1.25**	-3.20	-5.81 to -.60**
Widowed	-1.86	-6.51 to 2.77	.10	-5.13 to 5.34	-3.44	-6.93 to .04	-3.37	-5.96 to -.79**
Married	-	-	-	-	-	-	-	-
Duration of disease in years	-.06	-.25 to .13	-.16	-.38 to .04	-.09	-.24 to .04	-.06	-.17 to .03
Frequency of AlA	-.65	-1.14 -.16**	-.51	-1.06 to .04	-.67	-1.03 to -.30**	-0.38	-.65 to -.10**
Stage of disease								
Stage 2	-	-	-	-	-	-	-	-
Stage 3	-6.17	-9.49 to -2.85**	-6.73	-10.47 to -2.99**	-5.61-	-8.10 to -3.12**	-1.58	-3.42 to .26
Stage 4	-12.42	-17.33 to -7.50**	-9.15	-14.70 to 3.60**	-9.58	-13.27 to -5.89**	-2.34	-5.08 to .38
Stage 5	-13.72	-20.66 to -6.78**	-14.03	-21.86 to -6.20**	-13.09	-18.30 to -7.88**	-1.47	-5.33 to 2.38
Medical Rx								
Yes	.36	-2.87 to 3.60	-1.05	-4.70 to 2.60	.56	-1.86 to 2.99	-.33	-2.13 to 1.47
No	-	-	-	-	-	-	-	-
Comorbidity								
Yes	-3.20	-8.42 to 2.01	.91	-4.98 to 6.80	-1.42	-5.34 to 2.49	1.19	-1.70 to 4.10
No	-	-	-	-	-	-	-	-
Knowledge								
Good	-.47	-4.41 to 3.47	2.13	-2.32 to 6.58	-.47	-3.44 to 2.48	-.91	-3.11 to 1.27
Poor	-	-	-	-	-	-	-	-
Depression								
Yes	-1.17	-3.97 to 1.62	-.84	-4.00 to 2.31	-.16	-2.26 to 1.93	-.21	-1.77 to 1.34
No	-	-	-	-	-	-	-	-
Anxiety								
Yes	-1.76	-7.16 to 3.63	-1.05	-7.14 to 5.04	-4.29	-8.34 to -.238**	-1.59	-4.59 to 1.41
No	-	-	-	-	-	-	-	-
Social dysfunction								
Yes	-3.83	-7.70 to .03	2.00	-2.36 to 6.36	-1.29	-4.20 to 1.60	-.52	-2.67 to 1.62
No	-	-	-	-	-	-	-	-
Number of foot care per week	2.61	1.16 4.05**	4.90	3.27 to 6.53**	1.91	.82 to 2.99**	2.19	1.39 to 2.99**
Shoe wearing								
Yes	10.95	6.54 to 15.36**	6.26	1.28 to 11.23**	4.94	1.63 to 8.25**	1.20	-1.24 to 3.65
No	-	-	-	-	-	-	-	-
Wealth status								
Poor	-5.97	-10.25, -1.68**	-8.39	-13.23 to -3.55**	-6.56	-9.78 to 3.34**	-4.57	-6.95 to -2.19**
Medium	-2.41	-5.88 to 1.04	-3.96	-7.87 to -.05**	-3.14	-5.74 to -.54**	-1.05	-2.98 to .86
Rich	-	-	-	-	-	-	-	-
_cons	90.53	63.16 to 117.91	87.24	56.35, 118.13	88.27	67.71 to 108.82	38.77	23.55 to 53.99

AIA = Acute Adenolymphangitis Attacks,

** Variables statistically significant with p-values ≤ 0.05

Low HRQoL score of the physical domain was significantly associated with age (β = -.15, 95% CI = -.30 to -.01), family size (β = -1.25, 95% CI = -2.27 to -.22) being single (β = -8.60, 95% CI = -15.48 to -1.71), and frequency of ALA (β = -.65, 95% CI = -1.14 to -.16), and poor wealth status(β = -5.97, 95% CI = -10.25 to -1.68). Stage of disease was also significantly associated lower physical domain of HRQoL, Stage 3 (β = -6.17, 95% CI = -9.49 to -2.85), Stage 4 (β = -12.42, 95% CI = -17.33 to -7.50), Stage 5 (β = -13.72, 95% CI = -20.66 to -6.78) compared to stage 2. On the other hand, residing in rural areas (β = 12.17, 95% CI = 5.65 to 18.70), foot care (β = 2.61, 95% CI = 1.16 to 4.05), and shoe wearing (β = 10.95, 95% CI = 6.54 to 15.36) were associated with higher physical domain HRQoL. The linear regression model explained 64.12% the variance in physical domain of HRQoL.

The present analysis showed that, being single (β = -12.8, 95% CI -20.25), divorced (β = -5.90, 95% CI -11.28, to -0.69), stage 3 (β = -6.73, 95% CI = -10.47, to -2.99), stage 4 (β = -9.15, 95% CI = -14.70, to -3.60), stage 5 (β = -14.03, 95% CI = -21.86, to -6.20), poor wealth status (β = -8.39, 95% CI -13.23 to -3.55), medium wealth status (β = -3.96, 95% CI = -7.87 to -.05) were negatively associated variables with environmental HRQoL, whereas foot care (β = 4.90, 95% CI = 3.27, to 6.53 and shoe wearing (β = 6.54, 95% CI = 2.14, to 11.14) were positively associated with the environmental domain of HRQoL. About 58.94% of the variation in environmental domain of HRQoL was explained by these variables.

The analysis indicated that family size(β = -.97, 95% CI = -1.75 to -.21), being single(β = -11.90, 95% CI = -17.07, to -6.74 and divorced β = -4.77, 95% CI = -8.29, to -1.25) relative to married, frequency of ALA(β = -.67, 95% CI = -1.03 to -.30), the presence of anxiety(β = -4.29, 95% CI, -8.34, to -.238), poor wealth status (β = -6.56, 95% CI, -9.78, to—3.34) and medium wealth status(β = -3.14, 95% CI, -5.74, to -.54) relative to rich, were negatively associated with psychological domain of HRQoL. Similarly, the stage of diseases was found to affect psychological domain of HRQoL negatively, stage 3 (β = -5.61, 95% CI, -8.10 to -3.12), stage 4 (β = -9.58, 95% CI, -13.27 to -5.89), stage 5 (β = -13.09, 95% CI, -18.30 to -7.88) as compare to stage 2 patients. In contrast, the psychological domain of HRQoL was improved with residing in rural (β = 5.64, 95% CI = .74, to 10.55), frequent foot care (β = 1.91, 95% CI = .82, to 2.99), and shoe wearing (β = 4.94, 95% CI = 1.6 to 8.25). The linear regression model explained 71.19% the variance in psychological domain of HRQoL.

The predictors of the social domain of HRQoL were also analyzed. Accordingly, the regression model 52.97% of the variance in the social domain of HRQoL was explained by residence, foot care, and marital status, frequency of ALA, and wealth status. Residing in rural areas (β = 3.75, 95% CI = 11, to 7.38) and frequent foot care practice (β = 2.19, 95% CI = 1.39, to 2.99) were associated with the higher social domain of HRQoL. Being single ((β = -4.21, 95% CI = -8.04, to -.38) and divorced (β = -3.20, 95% CI = -5.81, to -.60) compared to married, frequency of an acute ALA (β = -.38 95% CI = -.65, to -.10) were negative predictors of social domain of HRQoL. Additionally, social domain of HRQoL score was lower among the poor wealth status participants (β = -4.57, 95% CI = -6.95, to -2.19).

## Discussion

The overall HRQoL of the study participants was higher than a study conducted in North Ethiopia [17]. In the study area there is implementation of community-based podoconiosis prevention and treatment programs [36] this might be the possible justification of the differences.

The present study showed that the social domain had the lowest score as compared to other domains. This result is supported by a study conducted from southern Ethiopia [24]. The possible justification of low social domain score might be the community avoiding physical contact with patients, excluding patients from social events like weddings and funerals, spitting on patients, pinching nose when walking pass patients at a distance; as result patients are dissatisfied with their relationships, feel guilty, hide and isolate themselves from the rest of the community members [24,25]. Social stigma reduces the social and psychological aspects of the quality of life of people with a podoconiosis [26].

The second-lowest score was observed in the psychological domain. This finding was similar to a study conducted in Cameroon with (PHQ-9; mean) score among people with lower-limb lymphoedema showed psychological and social domain of HRQoL were lower [18,23]. Studies documented that patient with podoconiosis do not enjoy and feel their life as meaningless didn’t accept their bodily appearance and experience negative feelings such as blue mood, despair, anxiety, depression, [18,32,37,38]. This might be the possible cause of the lowest score of psychological domain quality of life.

The environmental and physical domains had the highest score relative to social and psychological domains of HRQoL. This implies patients were satisfied with the availability of information that they needed in their day-to-day lives, satisfied with the conditions of their living place, getting medical treatment and apply foot care at home, ability to perform their daily living activities, and access to means of transportation might be the possible reason for the higher score of environmental and psychological domain quality of life.

In this study, stage of disease, frequency of ALA, and foot care, marital status, shoe-wearing and wealth status had a significant association with the three domains of quality of life and foot care, marital status, wealth status, and shoe wearing were significantly associated with all domain of quality of life.

As age increases the physical domain of HRQoL in the patients with podoconiosis decreases. This result is supported by the study conducted in Sri Lanka [30]. Similar findings were also reported on patients with chronic disease. This might be because of the physiological alteration of the patients as they got older. Older individuals are mostly limited on physical activities [39–41].

Our finding showed that patients who wear shoes had a better quality of life for the physical, environmental and psychological domains than patients who did not wear shoes. This result is supported by a study from Western Uganda [42]. Several studies suggested/recommended that consistent use of socks and shoes have a better outcome in the prevention and management of podoconiosis disease [43,44]. Podoconiosis is entirely avoidable if susceptible individuals wear socks and shoes that protect the skin against the irritant soil as well health education on foot care the cross-cutting issue to prevent and treat podoconiosis.

Marital status had a significant association with all domains of the HRQoL score. Those who were single and divorced were more likely to have poorer quality of life as compared to the married ones. This was supported by a study conducted in northern Ethiopia [17]. The possible explanation might be that married participants might have better social support from their spouses and relatives. In a qualitative study among podoconiosis, leprosy, and lymphatic filariasis patients, participants reports that they mostly relied on their children and spouses for support. They had got psychological and economical support, taking over household issues such as cooking and washing clothes. Additionally, the participants who live alone reported that they had no or very limited social supported [38].

Higher frequency of ALA was negatively associated with the physical, psychological, and social domain of health-related quality of life. Episodes of ALA have been reported as the most serious complications of podoconiosis; resulting in worsening disability and reduced quality of life [45,46]. Thus, lymphedema management is effective in improving clinical outcomes and the quality of life of people affected with podoconiosis [47,48]. Additionally, participants having podoconiosis related anxiety have the poor psychological domain of health-related quality of life.

In the present study, foot care was a positive predictor of HRQoL. Patients who had practiced frequent foot care had better HRQoL across all domains. This result was also supported by a study conducted from Western Uganda [42] that reported that podoconiosis patients who practice frequent foot care had low disease complications. Therefore, foot care prevents podoconiosis related foot ulcers [43,49], and acute attacks declined with consistent foot care and improve the quality of life and clinical stage of disease [46,50,51]. Besides this, foot care increases the patient’s sense of physical safety, decreases embarrassment, and enables patients to enjoy and participate in any social events. these further improve the patients’ overall quality of life.

Regarding residence, being a rural resident had a higher quality of life score in all domains of quality of life except in the environmental domain. This contradicts a study conducted in Northern Ethiopia [17].

This study showed that patients with stage 3, stage 4, and stage 5 had poor quality of life in respect to the physical, environmental and psychological domains than patients with stage two. This indicates that as the stage of the disease advanced, HRQoL decreased. This was congruent with a study conducted from Southern and Northern Ethiopia [17,45]. Increasing size and weight of the lower limbs makes it difficult or impossible in some cases to perform normal daily activities, in particular those related to standing and mobilizing. Impairments in body function and structure are intimately connected with an individual’s ability to perform essential activities. Individuals also faced difficulties participating in other important societal roles and events [16].

According to this finding patient with poor wealth status had lower quality of life scores in all domains of WHOQoL-BREF score as compared to patients with rich wealth status. This report is supported by the study conducted in Northern [17]. It is known that podoconiosis disease results in chronic disability and physical impairment. This has a significant impact on poor individuals’ ability to work or find employment, especially in rural areas where subsistence farming and manual labour are the major occupations. As a result, food insecurity was higher among podoconiosis patients [16,38,52]. On top of this, podoconiosis incur considerable out-of-pocket health payment on podoconiosis patients [53,54] such expenditure might prevent these patients from receiving appropriate medical care. Furthermore, participants with poor wealth status cannot fulfill their necessities.

### Limitation of the study

This study was a cross-sectional study that was only able to detect the association between the dependent variable with the predictor variables. So, failed in its ability to detect changes over time for some variables.

## Conclusion

Social and psychological domains of quality of life were lowest as compared to physical and environmental domains of quality of life. Stage of the disease, frequency of ALA, and presence of anxiety were significantly associated with lower quality of life for most of the domains. Whereas residence, shoe-wearing, and foot care were significantly associated with better quality of life of podoconiosis patients with all domains. Therefore, this study has several implications for podoconiosis prevention and treatment program. Health education on frequent foot care (hygiene, bandaging treatment) and shoe wearing for patients with podoconiosis, leads to physical improvement of the feet and legs, with decreased swelling and mossy lesions suggesting that high score of physical and environmental domains finally leads to improvement of overall quality of life score.

## Supporting information

S1 Abbreviations List of Abbreviations. (DOCX)
