# Supplementary material for: Health-related quality of life and associated factors among adult podoconiosis patients in Debre Elias district Northwest, Ethiopia
Source: PLoS Negl Trop Dis. 2022 Sep 2;16(9):e0010673. doi: 10.1371/journal.pntd.0010673 (PMC9477424; doi:10.1371/journal.pntd.0010673)
Supplement: S1 Abbreviations — (DOCX) [file pntd.0010673.s001.docx]

**List of Abbreviations**

ALA: Adeno Lymphangitis Attack; HRQoL: Health-Related Quality of Life; NTDs: Neglected Tropical Disease; WHO: World Health Organization; WHOQOL: World Health Organization Quality of Life; WHOQoL-BREF: World Health Organization Quality of Life Bref.
